# Antimicrobial Drug Resistance in *Corynebacterium diphtheriae mitis*

**DOI:** 10.3201/eid1711.110282

**Published:** 2011-11

**Authors:** Olivier Barraud, Edgar Badell, François Denis, Nicole Guiso, Marie-Cécile Ploy

**Affiliations:** Institut National de la Santé et de la Recherche Médicale, Limoges, France (O. Barraud, F. Denis, M.-C. Ploy);; University of Limoges, Limoges (O. Barraud, F. Denis, M.-C. Ploy);; Institut Pasteur, Paris, France (E. Badell, N. Guiso)

**Keywords:** bacteria, antimicrobial drug resistance, Class 1 integron, Corynebacterium diphtheria, antibiotic resistance, letter

**To the Editor:** *Corynebacterium diphtheriae* is the agent of pharyngeal and cutaneous diphtheria. We did a retrospective analysis of the antimicrobial drug susceptibilities of 46 *C. diphtheriae* isolates sent during 1993 through 2010 to the French National Reference Centre of Toxigenic Corynebacteria. The isolates came from metropolitan France and French overseas departments and territories. Only 1 isolate, *C. diphtheriae* biovar *mitis*, FRC24, expressed the following antimicrobial drug susceptibility profile: susceptible to penicillin, amoxicillin, ciprofloxacin, clindamycin, erythromycin, gentamicin, imipenem, kanamycin, rifampin, tetracycline, and vancomycin and resistant at an uncommonly high level to trimethoprim, sulfamethoxazole, and co-trimoxazole with Etest (bioMérieux, Marcy l’Etoile, France) MICs of >32, >1,024, and >32 mg/L, respectively.

This FRC24 isolate was isolated in 2008 from a cutaneous wound on a vaccinated 11-month-old child in Mayotte, an overseas department located in the Indian Ocean. Cutaneous carriage of *C. diphtheriae* is frequent in tropical countries where cutaneous diphtheria is endemic; cutaneous carriage represents a common mode of transmission of the bacterium. FRC24 was identified by using the API Coryne strip (bioMérieux). FRC24 is a toxigenic isolate; toxigenicity was confirmed by both *tox* gene detection and Elek test (*1*). Multilocus sequence typing was performed, and the sequence type (ST) of the isolate is ST91. This ST contains only this isolate and is part of lineage II, as are all *mitis* and *gravis* biovars (*2*).

To date, resistance to trimethoprim, sulfamethoxazole, or co-trimoxazole seems to be rare among the *C. diphtheriae* species, but few data are available (*3*). As trimethoprim resistance is often encoded by integron-driven *dfr* determinants, we looked for integrons. Integrons are bacterial genetic elements able to capture and express antimicrobial drug resistance gene cassettes (GCs) (*4*). GC movements are catalyzed by an integron-encoded integrase IntI. GCs, mainly promoterless, are usually expressed through a common Pc promoter (*5*). Only rare GCs contain their own promoter (*cmlA, qac, ereA1*). Three main classes of integrons have been described and are involved in the dissemination of antimicrobial drug resistance; class 1 is the most widely found in clinical isolates. Integrons have been mainly described among gram-negative bacteria; only a few studies have reported integrons in *Corynebacterium* spp (*6*,*7*).

After bacterial genomic DNA extraction (DNeasy Blood & Tissue Kit; QIAGEN, Courtaboeuf, France), a multiplex Taqman-based quantitative PCR approach able to detect the 3 main classes of integrons was performed (*8*). We found that FRC24 harbored a class 1 integron. Analysis of the GC array showed that this integron harbored 2 GCs: *dfrA16* of 588 bp conferring resistance to trimethoprim and *qacH* of 511 bp conferring resistance to quaternary ammonium compounds (GenBank accession no. FR822749). To our knowledge, this GC array has not been previously reported, even among reports of other gram-negative isolates. Moreover, a *qac* determinant has been found only once in a *Corynebacterium* species, *C. pseudogenitalium* (which harbors a *qacH* variant in the chromosome [GenBank accession no. ABYQ02000013]), but not in an integron background. GC arrays were followed by the *qacEΔ1* (which also confers resistance to quaternary ammonium compounds), *sul1* (resistance to sulfamethoxazole), and *orf5* determinants as found in most class 1 integrons (*4*). In class 1 integrons, 13 Pc variants have been described (*5*). In the FRC24 integron, the *dfrA16* expression was mediated through a strong Pc variant (PcW_TGN-10_) (*5*) that enables the high-level resistance observed for trimethoprim. As previously demonstrated, the *qacH* GC possessed its own promoter (*9*).

Trimethoprim is a commonly prescribed antimicrobial agent used in combination with sulfamethoxazole (co-trimoxazole) for the treatment of diarrheal diseases. This antimicrobial drug might have selected the emergence of such a strain expressing trimethoprim resistance. Furthermore, the FRC24 integron contains the antiseptic (quaternary ammonium compounds) resistance gene *qacH*. As cutaneous carriage of *C. diphtheriae* is frequent in tropical countries such as Mayotte, this bacterium could be exposed to quaternary ammonium compounds contained in disinfectants, hygienic hand washes, and cosmetic products. These products exert a selective pressure, which might play a role in selecting *qac*-containing strains, as has been suggested for *Staphylococcus* spp (*10*). For staphylococci, the MICs of quaternary ammonium compounds are >2 mg/L. With FRC24, we tested for the MIC of cetyltrimethylammmonium bromide and found a MIC of 4 mg/L, suggesting that *qacH* is expressed in FRC24.

The sequencing of the genetic environment of this integron showed that it was framed by 2 copies of the insertion sequence IS*6100* disrupting at the left-hand side the *intI1* integrase gene (Figure A1). IS*6100* has been described in a wide spectrum of host organisms, including *Corynebacterium* spp (*6*,*7*), thus enabling this integron to be efficiently transferred to various bacteria.

Our findings show that *C. diphtheriae* is able to harbor integrons, which is of clinical relevance. Indeed, this genetic feature would give the isolates the capacity to easily acquire new GCs, such as *ere* GCs encoding resistance to erythromycin, which is one of the antimicrobial drugs recommended for diphtheria treatment.

## Figures and Tables

**Figure A1:**
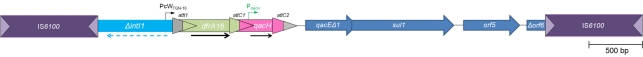
Physical map of the class 1 integron from *Corynebacterium diphtheriae* FRC24. Thin arrows represent coding sequences of *dfrA16* and *qacH* genes. Dashed arrow indicates the coding sequence of *intI1* disrupted by IS*6100*. The recombination sites *attI1*, *attC1*, and *attC2* are represented by triangles.
